# Long-term prognosis of clinically early IgA nephropathy is not always favorable

**DOI:** 10.1186/1471-2369-15-94

**Published:** 2014-06-19

**Authors:** Hajeong Lee, Jin Ho Hwang, Jin Ho Paik, Hyun Jin Ryu, Dong Ki Kim, Ho Jun Chin, Yun Kyu Oh, Kwon Wook Joo, Chun Soo Lim, Yon Su Kim, Jung Pyo Lee

**Affiliations:** 1 Department of Internal Medicine, Seoul National University Hospital, Seoul, Korea; 2 Department of Internal Medicine, Chung-Ang University Hospital, Seoul, Korea; 3 Department of Pathology, Seoul National University Bundang Hospital, Seongnam, Korea; 4 Department of Internal Medicine, Seoul National University Bundang Hospital, Seongnam, Korea; 5 Department of Internal Medicine, Seoul National University Boramae Medical Center, Seoul, Korea

**Keywords:** IgA nephropathy, Interstitial fibrosis, Progression of renal failure

## Abstract

**Background:**

The long-term prognosis of clinically early IgA nephropathy (IgAN) patients remains to be clarified. We investigated the long-term outcomes of IgAN patients with an apparently benign presentation and evaluated prognostic factors for renal survival.

**Methods:**

We included patients with biopsy-proven IgAN who had estimated glomerular filtration rates (eGFR) ≥60 mL/min/1.73 m^2^, normal blood pressure, and proteinuria <0.5 g/day at the time of biopsy. The primary outcome was progression to end-stage renal disease (ESRD). The secondary outcome was a 50% increase in serum creatinine level or an increase in proteinuria to >1 g/day.

**Results:**

The analysis included 153 patients who met the inclusion criteria. At diagnosis, their median systolic blood pressure was 120 (110–130) mmHg, eGFR was 85.9 (74.9–100.1) mL/min/1.73 m^2^, and proteinuria was 0.25 (0.13–0.38) g/day. Of these, 4 patients died and 6 reached ESRD. The 30-year renal survival rate was 85.5%. Three patients had increased serum creatinine levels and 11 developed proteinuria. Remission was observed in 35 (22.9%) patients. A moderate or severe degree of interstitial fibrosis (adjusted odd ratio [OR] 5.93, 95% confidence interval [CI] 1.44–24.45, *P* = 0.014) and hypoalbuminemia (adjusted OR 6.18, 95% CI 1.20–31.79, *P* = 0.029) were independent predictors of the secondary outcome.

**Conclusions:**

This study showed that the prognosis of early IgAN was not always favorable, even resulting in progression to ESRD in some cases. Hypoalbuminemia and interstitial fibrosis should also be considered important prognostic factors in clinically early IgAN patients.

## Background

IgA nephropathy (IgAN) is a common glomerular disease and an important cause of kidney failure. This disease accounts for more than half of all forms of primary glomerulonephritis in Korea [1]. Although dominant mesangial IgA deposits represent the diagnostic hallmark of IgAN, its clinical features are highly variable, ranging from simple hematuria with or without proteinuria to a rapidly progressive loss of renal function. Therefore, the renal survival and risk factors of long-term IgAN patients have been studied extensively over the last 30 years [2]. Previous studies indicate that the likelihood of dialysis or death can be estimated using three clinical risk factors: urinary protein excretion of more than 1 g/day, high blood pressure exceeding 140/90 mmHg, and a decreased estimated glomerular filtration rate (eGFR) of less than 60 mL/min/1.73 m^2^[3]. Among patients with all three risk factors, about 70–80% reached end-stage renal disease (ESRD) and 45% died within 30 years [4,5]. These clinical prognostic factors also independently predict a poor clinical course. However, information on the long-term outcomes of IgAN patients with a minor presentation is scarce.

Only a few studies have focused on long-term patient and renal outcomes in IgAN patients who had a renal biopsy performed for microscopic hematuria, normal renal function, and minimal proteinuria. Moreover, the results of previous studies are controversial. A recently published European cohort study found that IgAN patients who presented with minor urinary abnormalities and normal renal function did not progress to ESRD, and that more than one-third of patients achieved clinical remission [6]. Research performed in Hong Kong, however, found that 33% of patients with minimal proteinuria and preserved renal function developed more than 1 g/day of proteinuria [7,8]. In a cohort of Chinese IgAN patients with isolated microscopic hematuria who were followed for up to 12 years, a decrease in renal function was observed in 24% [9]. These studies suggest that IgAN patients require long-term follow-up due to the potential for progressive disease.

Our previously, reported findings on the mortality of IgAN patients, which were obtained from our long-term follow-up data [4], did not significantly differ from European data [10]. The present study focused on long-term outcomes and prognostic factors for renal survival in clinically early IgAN patients. We hypothesized that even patients presenting with benign manifestations would progress during long-term follow-up.

## Methods

### Ethics statement

Ethical approval was obtained from the Institutional Review Board of Seoul National University Hospital (H–1010–055–336), and this study was conducted in accordance with the principles of the Declaration of Helsinki. As the study was retrospective in design and did not involve any interventions, the Institutional Review Board waived informed consent for this study.

### Study subjects

**Figure 1:**
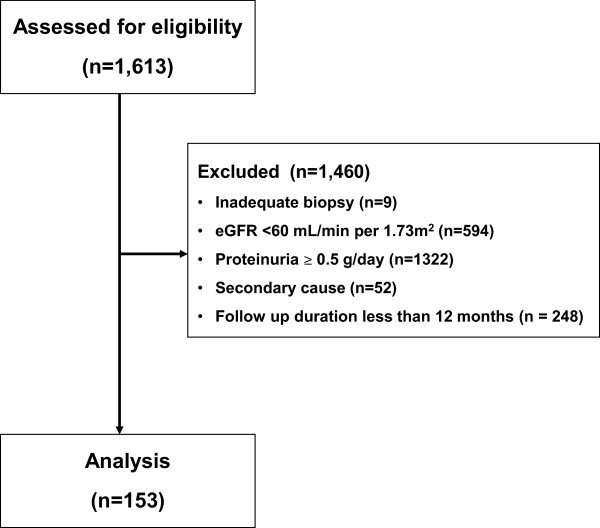
Participation flow diagram.

From 1979 to 2012, a total of 1,613 adult patients were diagnosed with IgAN based on immunofluorescence microscopy showing mesangial IgA deposition as the predominant or co-dominant immunoglobulin. We included IgAN patients with preserved renal function (eGFR ≥60 mL/min/1.73 m^2^) and minimal proteinuria (urinary protein-to-creatinine ratio <0.5 g/g creatinine or 24-h urinary protein <0.5 g/day). We excluded patients with fewer than 5 glomeruli per biopsy section and those in whom IgAN was the result of secondary causes, as indicated by clinical or laboratory evidence of systemic lupus erythematosus, Henoch-Schonlein nephritis, or liver cirrhosis. Patients who were followed for less than 12 months were also excluded. Ultimately, a total of 153 remaining patients were analyzed (Figure 1).

### Clinical parameters

Clinical information was collected from a review of computerized medical records. Demographic factors, including age, sex, body mass index, and blood pressure at the time of renal biopsy, were obtained. Blood and urine chemistry parameters were extensively reviewed from the time of renal biopsy to the time of last follow-up. Until 2010, serum creatinine levels were measured by Jaffe kinetic alkaline picrate method using a Hitachi 7600 analyzer (Toshiba, 200FR, Tokyo, Japan). Serum creatinine levels were re-calibrated to an isotope-dilution mass spectrometry assay (Roche diagnostic). The correction equation is as follows: recalibrated serum creatinine = 1.0734 x measured serum creatinine^(-0.2418)^. The eGFR was calculated using the Modification of Diet in Renal Disease equation [11,12]. Proteinuria was assessed using either a random urine protein to creatinine ratio (normal range, <0.2 g/g creatinine) or 24-h urinary protein measurement. Information on treatments prescribed during the follow-up period was collected, including exposure to immunosuppressive agents and to renin-angiotensin system (RAS) blockade by angiotensin-converting enzyme inhibitors or angiotensin receptor blockers.

### Pathological parameters

All native renal biopsies were processed according to light microscopy, immunofluorescence, and electron microscopy standard techniques. Histopathological changes were evaluated by, two pathologists who reviewed the renal biopsy slides. All IgAN biopsies were staged according to the WHO grading system [13]. The proportions of global sclerosis, segmental sclerosis, and crescent formation in the glomerular area were calculated using results obtained on light microscopy. Mesangial proliferation was also graded as none or minimal, mild, moderate, and severe. In the tubulointerstitial area, the degrees of tubular atrophy (TA), interstitial fibrosis (IF), and interstitial inflammation were graded semi-quantitatively as follows: none or minimal, mild, moderate, and severe.

### Outcome measurement

The primary outcome was ESRD progression or patient death. The start of the follow-up period was taken as the date of renal biopsy. ESRD was defined as progression to eGFR <15 mL/min/1.73 m^2^, initiation of permanent dialysis, or kidney transplantation. Data on mortality were obtained from the Korean National Statistical Office, and those on ESRD were collected from the Korean ESRD registry [14,15]. We combined all these data according to the unique identification number issued to all Koreans. The secondary outcome was renal progression, defined as a 50% serum creatinine increase or the development of proteinuria greater than 1 g/day. We also evaluated patient clinical remission, defined as the disappearance of microscopic hematuria confirmed on more than 3 occasions, proteinuria persistently lower than 0.2 g/day, and normal renal function.

### Statistical analysis

The data are presented as frequencies and percentages for categorical variables. Continuous variables are shown as medians and interquartile ranges (IQR). Comparisons between the outcome group and other groups were performed using the χ^2^ test for dichotomous variables and the Mann–Whitney test for asymmetric continuous variables. Cumulative renal survival was determined by the Kaplan-Meier method. Non-renal deaths were excluded from the renal survival rate analysis. The renal survival time for each patient was computed from the time of renal biopsy to the last follow-up. Associations between baseline variables and outcomes were tested using logistic regression. Potential confounding variables identified a priori included hypoalbuminemia (serum albumin level, <3.5 g/dL), RAS blockade, and pathological changes such as segmental sclerosis, interstitial fibrosis, and tubular atrophy. Variables that showed a significant association (*p* <0.10) in the univariate analysis or were of considerable theoretical relevance were entered into the multivariate stepwise logistic regression models. Analyses were performed using the SPSS software package (version 20.0, Chicago, IL, USA). All tests were two-tailed, with *P*-values <0.05 considered statistically significant.

## Results

### Baseline characteristics

Considering the exclusion criteria described above, a total of 153 (9.5%) patients were included in the final analyses. The median follow-up duration was 95 (38–207) months. Patient demographic information is summarized in Table 1. The age at the time of biopsy was 26 (20–36) years, and 43.8% of patients were male. The initial eGFR was 85.9 (74.9–100.1) mL/min/1.73 m^2^ and systolic blood pressure was 120 (110–130) mmHg. The median value of proteinuria was 0.25 (0.13–0.38) g/day. Approximately 43% of the patients were prescribed RAS blockers during the follow-up period. No patient was treated with immunosuppressive agents including corticosteroids.

**Table 1:** Baseline clinical characteristics of early IgAN patients

**Variables**	**Values**			
Age (years)	26 (20–36)			
Sex (male,%)	60 (43.8%)			
Body mass index (kg/m^2^)	21.2 (20.0–23.5)			
Systolic blood pressure (mmHg)	120 (110–130)			
Serum creatinine (mg/dL)	0.90 (0.80–1.09)			
eGFR (mL/min/1.73 m^2^)	85.9 (74.9–100.1)			
Proteinuria (g/day)	0.25 (0.11–0.38)			
Serum cholesterol (mg/dL)	159 (144–185)			
Serum albumin (g/dL)	4.1 (3.9–4.4)			
Plasma hemoglobin (g/dL)	13.2 (12.3–14.4)			
Serum IgA (mg/dL)	302 (223–417)			
Follow up duration (months)	95 (38–207)			
RAS blockade (N,%)	66 (43%)			
Glomerulus number	37 (20–58)			
Global sclerosis (%)	4.0 (0–11.0)			
Segmental sclerosis (%)	0 (0–5.0)			
Crescent formation (yes,%)	14 (9.2%)			
WHO grade	I	II	III	IV
	19 (12.4%)	84 (54.9%)	37 (24.2%)	10 (6.5%)
	None	Mild	Moderate	Severe
Mesangial proliferation (yes,%)	16 (10.7%)	114 (74.5%)	16 (10.7%)	4 (2.6%)
Tubular atrophy (yes,%)	43 (28.1%)	85 (55.6%)	19 (12.4%)	2 (1.3%)
Interstitial fibrosis (yes,%)	30 (19.6%)	100 (65.4%)	17 (11.1%)	2 (1.3%)
Interstitial inflammation (yes,%)	38 (24.8%)	110 (71.9%)	1 (0.7%)	0 (0.0%)

Continuous variables are presented by median (interquartile range). Categorical variables are presented by frequency (percentage). eGFR, estimated glomerular filtration rate; RAS, renin–angiotensin system.

In the histopathological review, more than half of the patients showed minimal or mild pathological change while 47 (30.7%) patients had advanced pathological change, with WHO grade higher than III. Glomerular crescent formation was found in 9.2% of the patients. Moreover, more than 12% of patients had a moderate to severe degree of mesangial proliferation, TA or IF.

### Primary outcomes

**Figure 2:**
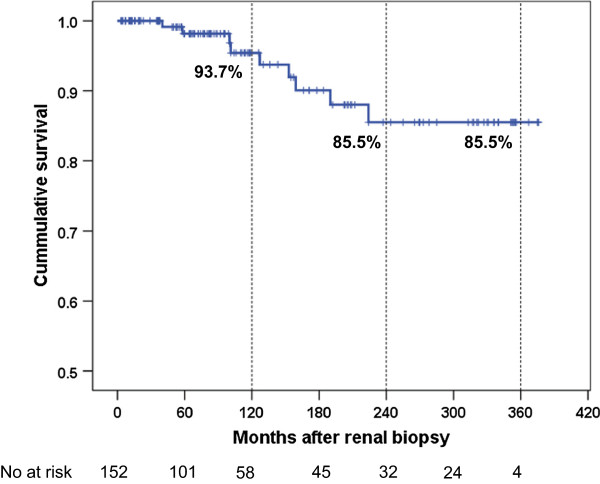
**Cumulative renal and patient survival after kidney biopsy in early IgAN patients.** The primary endpoint is free of death or end stage renal disease. The number of patients remaining at 60, 120, 180, 240, 300 and 360 months of follow–up are shown at the bottom.

During the observation period, 6 patients developed ESRD and 4 patients died. The 30-year renal survival rate was 85.5% (Figure 2). The details of patients who reached to ESRD are described in Table 2. Although they had preserved renal function and minimal proteinuria at the time of biopsy, half of the patients had an advanced pathological grade relative to their clinical manifestation. Two patients had segmental sclerosis, and one had moderate degree of TA/interstitial inflammation. None of the patients who reached ESRD showed crescent formation or vascular structural change. Time to ESRD ranged from 5 to 19 years. Four patients died. One patient died subsequent to ESRD progression. However, the remaining 3 patients died with functioning kidneys. The patient in whom renal failure progressed to ESRD 153 months following kidney biopsy, died 142 months after peritoneal dialysis was initiated. The cause of death was colonic pseudo-obstruction and intestinal perforation. Causes of death in the patients who had died with functioning kidneys were traffic accident in one case, ovarian cancer in one case, and unknown in the other cases.

**Table 2:** Clinical manifestation of patients who reached to end–stage renal disease

	**Patient A**	**Patient B**	**Patient C**	**Patient D**	**Patient E**	**Patient F**
Body mass index (kg/m^2^)	24.6	Unknown	18.3	21.6	23.2	21.4
Blood pressure (mmHg)	110/10	130/90	100/70	110/70	110/70	100/60
Blood urea nitrogen (mg/dL) [10-26]	9	17	12	12	12	NA
Serum creatinine (mg/dL) [0.70–1.40]	1.00	1.20	0.90	1.20	0.70	1.30
Creatinine clearance (mL/min)	78.5	NA	84	87	NA	66
eGFR (mL/min/1.73 m^2^)	72.1	74.1	77.1	82.7	152.4	74.6
Serum albumin (g/dL) [3.3–5.2]	4.6	4.9	3.5	4.4	4.0	NA
Proteinuria (g/day)	0.21	0.22	0.35	0.34	0.18	0.20
WHO grade	III	II	II	III	III	NA
Global sclerosis (N/Glom No)	0/19	1/15	0/8	2/32	1/35	0/5
Segmental sclerosis (N/Glom No)	0/19	0/15	0/8	2/32	2/35	0/5
Crescent formation (N/Glom No)	0/19	0/15	0/8	0/32	0/35	0/5
Mesangial proliferation	None	Mild	None	Mild	Mild	Moderate
Tubular atrophy	Mild	None	None	Moderate	Mild	NA
Interstitial fibrosis	None	None	None	Mild	Mild	NA
Interstitial inflammation	Mild	None	Mild	Moderate	None	NA
Vascular change	None	None	None	None	None	None
Time to ESRD (months)	153	224	101	100	58	159

Continuous variables are presented by median (interquartile range). Categorical variables are presented by frequency (percentage). eGFR, estimated glomerular filtration rate; RAS, renin–angiotensin system.

### Secondary outcomes

Secondary outcomes could be analyzed in 118 of the 153 patients, for whom renal function and urinalysis data were available. Three patients showed a greater than 50% increase of serum creatinine levels compared with those at baseline. Eleven patients developed proteinuria with protein levels of at least 1.0 g/day during the follow-up period. The baseline clinical characteristics of progressive patients were similar to those of stable patients (Table 3). However, patients who met the secondary outcome had a higher frequency of segmental sclerosis (35.7% vs 8.7%, *P* = 0.012), IF of more than a moderate degree (35.7% vs 7.8%, *P* = 0.009), and RAS blockade use (84.6% vs 45.4%, *P* = 0.009).

**Table 3:** Comparison between patients with secondary renal outcome and those without

**Variables**	**Renal outcome (-)**	**Renal outcome (+)**	***P*–value**
N	104	14	
Age at the time of biopsy (years)	26 (19–37)	27 (25–35)	0.640
Sex (male, %)	47/104 (45.2%)	3/14 (21.4%)	0.148
Body mass index (kg/m^2^)	21.6 (19.9–23.5)	21.7 (20.5–23.1)	0.749
Systolic blood pressure (mmHg)	120 (110–126)	115 (110–130)	0.990
Serum creatinine (mg/dL)	0.90 (0.80–1.04)	0.80 (0.70–0.90)	0.016
eGFR (ml/min/1.73 m^2^)	86.4 (74.9–105.6)	93.0 (82.5–113.2)	0.142
Albumin (g/dL)	4.1 (3.9–4.4)	3.9 (3.5–4.3)	0.038
Proteinuria (g/day)	0.25 (0.10–0.40)	0.28 (0.10–0.41)	0.680
WHO grade	2.0 (2.0–2.0)	2.5 (2.0–3.0)	0.047
Global sclerosis ≥ 10%	32/104 (30.8%)	8/14 (57.1%)	0.214
Segmental sclerosis ≥ 10%	9/104 (8.7%)	5/14 (35.7%)	0.012
Cresent	10/104 (9.6%)	1/14 (7.1%)	1.000
Mesangial proliferation	15/103 (14.6%)	3/14 (21.4%)	0.450
Interstitial fibrosis	8/103 (7.8%)	5/14 (35.7%)	0.009
Interstitial inflammation	1/103 (1.0%)	0/14 (0.0%)	1.000
Tubular atrophy	11/103 (10.7%)	5/14 (35.7%)	0.024
RAS blockade (N, %)	48/103 (45.4%)	12/14 (84.6%)	0.009

### Predictors of outcome

Table 4 shows the results of predictor analyses according to secondary and composite outcomes. For the primary outcome, we failed to identify any clinical or pathological risk factors. In the univariate analysis, hypoalbuminemia < 3.5 g/dL, global and segmental sclerosis, IF, TA, and RAS blockader use were identified as potential predictors of the secondary outcomes. After the multivariate analysis, only hypoalbuminemia (odds ratio [OR] 11.89, 95% CI 2.10–67.23, *P* = 0.005) and IF (OR 5.93, 95% CI 1.44–24.45, *P* = 0.014) remained as independent risk factors. Regression analyses of a composite of the primary and secondary outcomes showed similar results to analyses of the secondary outcome. Both hypoalbuminemia (OR 6.18, 95% CI 1.20–31.79, *P* = 0.029) and IF (OR 3.82, 95% CI 1.11–13.11, *P* = 0.033) remained independent determinants of the composite outcome.

**Table 4:** Univariate and multivariate logistic regression analysis for development of outcome

**Variables**	**Wald**	**OR (95% CI)**	***P*–value**
***Secondary outcome***			
Univariate			
Hypoalbuminemia	9.68	13.33 (2.61–68.19)	0.002
Global sclerosis	3.58	3.00 (0.96–9.36)	0.058
Segmental sclerosis	7.23	5.86 (1.62–21.29)	0.007
Interstitial fibrosis	7.97	6.60 (1.78–24.45)	0.005
Tubular atrophy	5.72	4.65 (1.32–16.37)	0.017
Use of RAS blockade	5.97	6.88 (1.47–32.27)	0.015
Multivariate analysis			
Hypoalbuminemia	7.85	11.89 (2.10–67.23)	0.005
Interstitial fibrosis	6.06	5.93 (1.44–24.45)	0.014
***Composite outcome***			
Univariate			
Hypoalbuminemia	5.99	7.19 (1.48–34.86)	0.014
Segmental sclerosis	2.89	2.84 (0.85–9.47)	0.089
Interstitial fibrosis	5.85	4.31 (1.32–14.09)	0.016
Tubular atrophy	3.72	3.03 (0.98–9.47)	0.054
Multivariate analysis			
Hypoalbuminemia	4.75	6.18 (1.20–31.79)	0.029
Interstitial fibrosis	4.53	3.82 (1.11–13.11)	0.033

Continuous variables are presented by median (interquartile range). Categorical variables are presented by frequency (percentage). eGFR, estimated glomerular filtration rate; RAS, renin–angiotensin system.

### Clinical remission

During follow-up, 36 (31.0%) patients showed loss of proteinuria or microscopic hematuria along with stable blood pressure and renal function, in other words, clinical remission. Patients who achieved clinical remission had low systolic blood pressures (median [IQR], 114 [110–124] mmHg vs 120 [110–120] mmHg), less TA (11.4% vs 12.5%) and less IF (8.6% vs 11.3%). However, these differences were all statistically non-significant. Only the amount of proteinuria (0.28 [0.13–0.41] vs 0.21 [0.09–0.33], *P* = 0.021) and prescription of RAS blockade (34.3% vs 56.3%, P = 0.042) differed significantly according to clinical remission. Our regression analysis did not find any predictors of clinical remission (data not shown).

## Discussion

The clinical course of IgAN is highly variable. However, it is essential to determine whether a patient is at high risk for renal insufficiency in order to establish an individualized management plan. Forecasting the prognosis of benign IgAN on the basis of the currently known prognostic factors or modeling systems is challenging. Experience gathered over a sufficient observation period is essential to address these patients’ renal survival. Based on our long follow-up period, we demonstrated that even clinically early IgAN patients can show a progressive disease trajectory in Korea. More than moderate degree of IF and hypoalbuminemia were independent predictors of renal progression. This study is uniquely placed to clarify the prognosis of clinically early IgAN.

Our results are similar to those of previous Chinese and Japanese studies [8,9,16,17], and are contrary to those of a recent European study [6] (Table 5). In particular, our results evoke awareness of the fact that, even with a clearly benign initial presentation, IgAN patients can have a malignant renal or patient outcome. In this cohort, one in every 25 early IgAN patients progressed to ESRD during their lifetimes. Such an observation has not been reported previously. Considering that baseline characteristics among previous studies were similar, the cause of the varying prognosis of clinically early IgAN requires investigation. One possible explanation is the difference in the definition of outcomes. In the Chinese studies, renal progression was defined only in terms of worsening and not in relation to initial renal function [8,9]. Conversely, the European study defined renal progression as an increase in serum creatinine levels of ≥50% from initial levels or ESRD progression. However, our study separated ESRD progression and impaired renal function, and our definition of impaired renal function was derived from the European study. Our data indicate that the prognosis of early IgAN patients is relatively worse than that of similar European IgAN patients even when a comparable outcome definition is applied.

**Table 5:** Comparisons of definition and outcome of early IgAN with the previous studies

	**Hong Kong (‘00)**	**Hong Kong (‘01)**	**China (‘08)**	**Europe (‘12)**	**Korea (‘14)**
N	45 (NA)	72 (10%)	177 (NA)	141	153 (8.8%)
Definition	Cr < 1.3 mg/dL	Cr < 120 μmol/L	eGFR > 90 mL/min	eGFR > 60 mL/min	eGFR > 60 mL/min
	Proteinuria < 0.4 g/day	Proteinuria < 0.4 g/day	Proteinuria < 0.4 g/day	Proteinuria < 0.5 g/day	Proteinuria < 0.5 g/day
	HTN (-)	HTN (-)	HTN (-)		HTN (-)
FU months	123 (60 –180)	84 (14–112)	111 (109–205)	108 (60– 180)	95 (38–207)
Age (years)	29 (15–57)	27 (15–50)	38 ± 16	23 (5–71)	26 (16–65)
Outcome					
ESRD	No	1 patient	No	No	6 patients
Cr ↑	6 (13%)	5 (7%)	43 (24%)	5 (3.5%)	3 (2.1%)
Proteinuria ↑	15 (34%)	24 (33%)	79 (46%)	21 (14.9%)	11 (18.3%)
HTN	11 (24%)	19 (26%)	68 (38%)	23 (16.3%)	NA
Remission	NA	10 (14%)	10 (6%)	53 (38%)	36 (25%)

NA, not applicable; Cr, creatinine; eGFR, estimated glomerular filtration rate; HTN, hypertension; ESRD, end stage renal disease.

The divergence in findings may also be explained by racial differences. Recent publications suggest that an Asian racial origin could be identified as a risk for disease progression in IgAN [18,19]. Despite the fact that our study and the European study enrolled early IgAN patients with similar baseline renal function and proteinuria levels and used similar outcome definitions, renal prognosis in our cohort was considerably different from that observed in the European cohort. To clarify the influence of ethnicity on these observations, a delicate genetic analysis with consideration of phenotype should be performed.

Lead-time bias may also explain the different outcomes in early IgAN. Because of variations in biopsy practices, the disease is detected at different times in its natural course. Even among early IgAN patients with similar initial presentations, the duration of disease could be different. Some patients visit the clinic immediately after gross hematuria or incidentally detected hematuria, while others visit several years after the initial manifestation. However, clinicians can only conduct assessments at the time of initial visit or biopsy. Therefore, a comparison of these patients using only initial data could be limited by lead-time bias.

Our data also showed that pathological changes including IF can be important for renal risk prediction in clinically early IgAN patients. More than 12% of patients showed a more than moderate degree of tubulo-interstitial changes. Furthermore, IF is an independent predictor of ESRD progression in this study. Such results are consistent with some previous studies [9,20,21] and inconsistent with another study [6]. Although IgAN is a glomerular disease, tubulo-interstitial injury via the mesangio-podocytic-tubular crosstalk plays an important role in mediating renal fibrosis and, ultimately resulting in, renal failure [22]. In our study, mesangial hypercellularity or interstitial inflammation was not associated with renal progression. Those are considered relatively early renal injury markers, whereas IF is regarded as relatively advanced marker in IgAN patients with minor abnormalities. Indeed, IF is one component of the Oxford classification, although it is validated mostly in patients with proteinuria of more than 1 g/day [23,24]. Our study results support the applicability of the Oxford classification even in IgAN patients with a minimal clinical presentation.

We further demonstrated that hypoalbuminemia is a significant predictor of renal outcome. These finding is consistent with those in previous IgAN studies [4,25] as well as those in other CKD studies [26,27]. Lower serum albumin levels can be explained by the amount of proteinuria, nutritional status or combined inflammation. In our cohort, 7 patients showed reduced serum albumin levels. Their proteinuria amount was ranged between 0.16 and 0.45 g/day. Three patients had combined inflammation, and one patient suffered from tuberculosis and was subsequently malnourished. In other words, hypoalbuminemia may contribute to a poor renal outcome, independently from proteinuria.

The particular strengths and insights gained from this investigation include the long follow-up duration and large sample size, which is specifically important in IgAN research because of the insidious course of this disease. We were able to assess hard outcomes such as ESRD progression and mortality in this study. Moreover, we were able to suggest racial differences in IgAN prognosis by using baseline characteristics and outcome definitions similar to those used in the prior European study. We were also able to alarm many nephrologists and primary physicians who have managed clinically early IgAN patients with ease, especially in Korea.

However, several shortcomings remain to be resolved. First, the number of outcomes observed in our cohort was too small despite the long follow-up duration. Lack of events hampered our ability to perform robust multivariate predictive modeling. Second, this is a single-center, retrospective study, and therefore, we could not take into account diverse management strategies according to individual clinicians. Although hard outcome data were collected by both medical record review and from a national registry or statistics, the last clinical status remains unknown in some cases. Third, we could not use the Oxford classification in this study. However, we were able to measure each component of the Oxford classification semi-quantitatively, as shown in Table 1. Lastly, we could not clarify the precise reason for progression to ESRD among patients with initially benign clinical presentations. We proposed different outcome definitions, lead-time bias, and ethnicity as potential explanations. However, we could not prove these, and further sequential investigation is therefore warranted.

## Conclusions

In this study, we demonstrated that clinically early IgAN does not always show a favorable outcome and may even progress to ESRD. Patients with IF and hypoalbuminemia should be more aggressively monitored, especially in Korea.

## Abbreviations

IgAN: IgA nephropathy; eGFR: Estimated glomerular filtration rate; ESRD: End-stage renal disease; RAS: Renin-angiotensin system; TA: Tubular atrophy; IF: Interstitial fibrosis; IQR: Interquartile ranges; OR: Odds ratio; CI: Confidence interval.

## Competing interest

The authors declare that they have no competing interests.

## Authors’ contributions

All authors contributed extensively to the work presented in this paper at all stage. HL, HJC and JPL conceived the design of this research and wrote the manuscript. JPL supervised this project. KWJ and YKO assembled input data. JHB and JHH performed statistical analyses. HJR and CSL interpreted the data analyses. DKK and YSK gave conceptual advice and commented on the manuscript. All authors read and approved the final manuscript.

## Pre-publication history

The pre-publication history for this paper can be accessed here:

http://www.biomedcentral.com/1471-2369/15/94/prepub
